# Cervical amputation versus vaginal hysterectomy: a population-based register study

**DOI:** 10.1007/s00192-016-3119-0

**Published:** 2016-08-16

**Authors:** Ida Bergman, Marie Westergren Söderberg, Anders Kjaeldgaard, Marion Ek

**Affiliations:** 1 Department of Clinical Science and Education, Södersjukhuset, Karolinska Institutet and the Division of Obstetrics and Gynecology at Södersjukhuset, Stockholm, Sweden; 2 Department of Clinical Sciences, Karolinska Institutet, Stockholm, Sweden

**Keywords:** Anterior colporrhaphy, Cervical amputation, Pelvic organ prolapse, Recurrence, Uterine prolapse, Vaginal hysterectomy

## Abstract

**Introduction and hypothesis:**

Surgical management of uterine prolapse varies greatly and recently uterus-preserving techniques have been gaining popularity. The aim of this study was to compare patient-reported outcomes after cervical amputation versus vaginal hysterectomy, with or without concomitant anterior colporrhaphy, in women suffering from pelvic organ prolapse.

**Method:**

We carried out a population-based longitudinal cohort study with data from the Swedish National Quality Register for Gynecological Surgery. Between 2006 and 2013, a total of 3,174 patients with uterine prolapse were identified, who had undergone primary surgery with either cervical amputation or vaginal hysterectomy, with or without concomitant anterior colporrhaphy. Pre- and postoperative prolapse-related symptoms and patient satisfaction were assessed, in addition to complications and adverse events. Between-group comparisons were performed using univariate and multivariate logistic regression.

**Results:**

There were no differences between the two groups in neither symptom relief nor patient satisfaction. In both groups a total of 81 % of the women reported the absence of vaginal bulging 1 year after surgery and a total of 89 % were satisfied with the result of the operation. The vaginal hysterectomy group had a higher rate of severe complications than the cervical amputation group, 1.9 % vs 0.2 % (*p* < 0.001). The vaginal hysterectomy group also had a longer duration of surgery and greater perioperative blood loss, in addition to longer hospitalization.

**Conclusions:**

Cervical amputation seems to perform equally well in comparison to vaginal hysterectomy in the treatment of uterine prolapse, but with less morbidity and a lower rate of severe complications.

## Introduction

Pelvic organ prolapse (POP) is a common condition amongst the female population and the lifetime risk for prolapse or incontinence surgery is 11 % by the age of 80 years, with a 29 % risk of reoperation [1]. Surgical management of uterine prolapse varies greatly and there are currently insufficient data to guide practice [2]. Vaginal hysterectomy, including some kind of vaginal vault suspension, has traditionally been the most common surgical procedure in the treatment of uterine prolapse [1, 3], but uterus-preserving techniques are gaining popularity [4, 5]. Recent studies have shown a desire from the patients to retain the uterus in the case of equal outcome with hysterectomy [6, 7]. Uterus-preserving techniques have been shown to be associated with less morbidity and shorter hospitalization [8] and it has also been suggested that hysterectomy causes damage to the vascular and nerve supply of the pelvis, resulting in bladder dysfunction and recurrence of prolapse [9, 10]. Procedures such as abdominal sacrocolpopexy and sacrospinous fixation have been evaluated in randomized trials and have shown high success rates in comparison to vaginal hysterectomy [2, 11, 12]. Randomized trials evaluating techniques which include amputation of the cervix are lacking. In some papers cervical amputation is even described as an obsolete procedure [12]. However, based on the current literature, procedures including amputation of the cervix appear to be equally as effective with regard to anatomical cure and recurrence rates, but with less morbidity than vaginal hysterectomy [8, 13–15].

The aim of the present study was to compare the outcomes of cervical amputation versus vaginal hysterectomy, with or without concomitant anterior vaginal wall repair, in women suffering from uterine prolapse, using a population-based cohort selected from a national quality register.

## Materials and methods

### Data source

In this longitudinal cohort study data were collected from the Swedish National Quality Register for Gynecological Surgery. The register was established in 1997 and the majority (44 out of 55) of Sweden’s gynecological departments participate. Collection of data concerning POP surgery started in 2006. Data are collected prospectively using patient questionnaires and forms completed by the physicians. The physicians report data on admission, surgery, and discharge, including preoperative gynecological examination of the patient. The preoperative patient questionnaires include questions concerning demographic data and medical history in addition to questions about symptoms associated with POP. Postoperative questionnaires are filled in 8 weeks and 1 year after surgery and include questions about POP symptoms, complications, and patient satisfaction. The study design, including the use of data from the register, was approved by the Regional Board of Ethics in Stockholm, Sweden (Dnr 2014/958-31/4).

### Study population

The patients included in the study were women with symptomatic uterine prolapse stage I–IV, with or without anterior vaginal wall prolapse stage I–IV, according to the validated Pelvic Organ Prolapse Quantification System (POP-Q) [16], who have undergone either cervical amputation or vaginal hysterectomy, with or without concomitant anterior colporrhaphy, between January 2006 and December 2013. Exclusion criteria included previous prolapse or urinary incontinence surgery, a uterus larger than 12 weeks’ gestation, and concomitant posterior colporrhaphy or enterocele repair. A total of 4,047 patients, meeting the criteria described above, were identified in the register database.

### Description of the procedures

In the case of concomitant anterior colporrhaphy, this procedure is performed first. Anterior colporrhaphy involves a transvaginal incision of the anterior vaginal wall, 2–3 cm posterior to the external urethral meatus almost to the vaginal vault or neck of the cervix, and dissection of the bladder from the vagina. The pubocervical fascia is then adapted in the midline, after which the vaginal epithelium is closed.

In cervical amputation, the cervix is circumcised and the bladder is dissected from the cervical neck. The peritoneum is not opened. After amputation of the cervix, the vaginal epithelium and the cardinal ligaments are often sutured to the stump.

In vaginal hysterectomy the vaginal wall around the cervix is circumcised. After bladder dissection, the anterior and posterior peritoneum is opened. The uterus is released in several steps using clamps and sutures or an electro-thermal bipolar tissue sealing and dividing system. Vault suspension is often performed by suturing the uterosacral or cardinal ligaments to the vaginal vault. The mucosa is then closed.

### Outcomes

Baseline data were collected both from forms completed by the physician (age, functional status, and POP-Q) and from the preoperative questionnaire completed by the patient (BMI, parity, smoking, cesarean deliveries, use of estrogen replacement therapy, and menopausal status). Functional outcome measures were retrieved from the questionnaires completed by the patients. The primary outcome was patient-reported vaginal bulging at the 1 year follow-up questionnaire: “Do you experience a feeling of bulging or protrusion in the vaginal area?” The question was dichotomized from five answer options (never or almost never into “no” and 1–3 times per month, 1–3 times per week, and daily into “yes”). Secondary outcomes included surgical complications and adverse events related to the procedure reported by the physician, patient-reported satisfaction, changes in urinary and bowel symptoms, and sexual function 1 year after surgery. Questions about urinary and bowel symptoms were recoded from five answer options into three (never and almost never into “never,” 1–3 times per month, 1–3 times per week into “sometimes,” and daily into “daily”). Sexual activity was reported by answering the question “Have you had coitus in the past 3 months?” Data on complications were reported by the physician at the time of surgery and up to 1 year postoperatively. The severe complications included severe intra-abdominal bleeding, severe intra-abdominal infections or sepsis, ureteric injuries, bowel injuries, myocardial infarction, and severe complications related to the anesthesia. The mild to moderate complications consisted of bladder injuries, lower urinary tract infections or other mild postoperative infections, obstipation, nausea, and prolonged postoperative pain, vaginal bleeding, or fatigue.

### Statistical analysis

Baseline characteristics are presented as means ± SD or medians and range for continuous variables and as frequencies for categorical variables. For the comparison of baseline variables between groups we used the Mann–Whitney *U* test when analyzing continuous data and Fisher’s exact test for categorical variables. Categorical endpoints were analyzed using univariate and multivariate logistic regression, dichotomized outcomes with binary regression and multiple answer outcomes with the proportional odds model. Outcome measures were analyzed using logistic regression. The regression model adjusted for the following variables: surgical method, age, parity, body mass index (BMI), preoperative degree of anterior and apical compartment prolapse (according to the POP-Q), concomitant anterior colporrhaphy, symptoms at baseline, and menopausal status. Results of the logistic regression analyses are presented as odds ratios with 95 % confidence intervals. Comparison of outcomes within groups were analyzed using the Wilcoxon signed rank and McNemar tests. A *p* value of <0.05 was considered significant for all comparisons. Statistical analyses were performed using SPSS version 22 software (IBM, Armonk, NY, USA).

## Results

### Study population

**Fig. 1:**
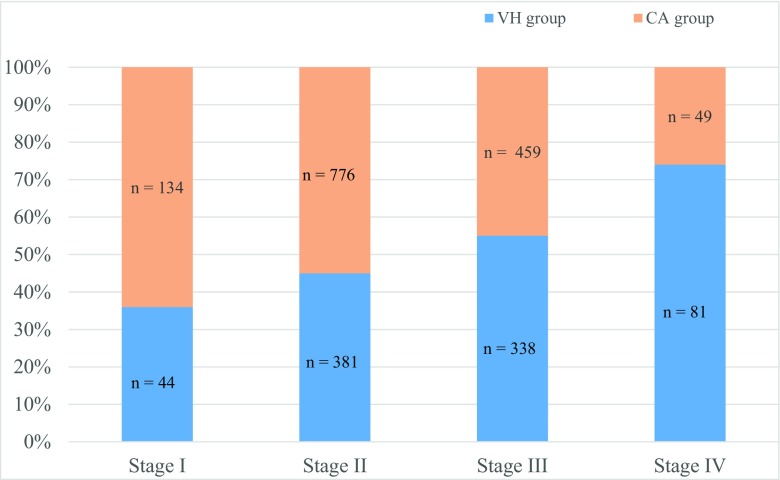
Number of vaginal hysterectomies (VH) in relation to cervical amputations (CA) at different stages of uterine prolapse

Of the 4,047 patients eligible for the study, a total of 3,174 (78 %) women responded to the primary outcome question (sensation of vaginal bulging) in the 1-year follow-up questionnaire. Of the 3,174 respondents, 1,195 patients (38 %) were treated with vaginal hysterectomy and 1,979 patients (62 %) with cervical amputation. There were no significant differences in baseline characteristics between the groups, except for menopausal status (Table 1). Concomitant anterior colporrhaphy was performed in 1,013 patients in the hysterectomy group (85 %) and in 1,757 patients in the cervical amputation group (89 %; Table 1). The relative number of cervical amputations in comparison to vaginal hysterectomies was greater in stage I–II apical descent, whereas the relative number of hysterectomies was higher in stage III–IV (Fig. 1).

**Table 1:** Baseline characteristics of the 3,174 study patients

Characteristic	VH group (*n* = 1,195)	CA group (*n* = 1,979)	*p* value
Age at surgery (years) mean (±SD)	63.2 (±10.4)	63.1 (±10.5)	1.0
Body mass index (kg/m^2^) mean (±SD)	25.9 (±4.1)	25.8 (±3.8)	0.6
Parity median (range)	2 (0–10)	2 (0–10)	0.1
Cesarean sections median (range)	0 (0–3)	0 (0–3)	0.4
Current smoker, *n* (%)			
Yes	118 (11)	158 (10)	0.3
No	960 (89)	1,461 (90)	
Estrogen replacement therapy, *n* (%)			
Yes	67 (6)	87 (5)	0.4
No	1,049 (94)	1,591 (95)	
Postmenopausal, *n* (%)			
Yes	800 (77)	1,284 (81)	0.03
No	236 (23)	307 (19)	
ASA classification, *n* (%)			
I	699 (58)	1,203 (61)	0.5
II	464 (39)	731 (37)	
III	32 (3)	44 (2)	
POP-Q stage apex, *n* (%)			
I	44 (5)	134 (9)	
II	381 (45)	776 (55)	<0.001
III	338 (40)	459 (32)	
IV	81 (10)	49 (4)	
POP-Q stage anterior wall, *n* (%)			
I	38 (5)	89 (6)	<0.001
II	351 (43)	716 (52)	
III	359 (44)	536 (39)	
IV	75 (9)	47 (3)	
Concomitant anterior colporrhaphy, *n* (%)			
Yes	1,013 (85)	1,757 (89)	0.001
No	181 (15)	222 (11)

### Outcome measures

Table 2 describes the mechanical, urinary and bowel symptoms, and sexual activity before and 1 year after surgery within the treatment groups. One year postoperatively, there was significant relief from all urinary and bowel symptoms in both treatment groups. Sexual activity also improved significantly in both groups. Comparisons between vaginal hysterectomy and cervical amputation 1 year after surgery are shown in Table 3. There were no statistical differences between the two groups neither in symptom relief nor in patient satisfaction. In both groups a total of 81 % of the women with preoperative vaginal bulging were asymptomatic 1 year after surgery and a total of 89 % were satisfied with the result of the operation. The results persisted after multivariate logistic regression analysis.

**Table 2:** Comparison of patient-reported symptoms within groups

Symptoms	VH group (*n* = 1,195)			CA group (*n* = 1,979)		
	Preoperatively, *n* (%)	Postoperatively, *n* (%)	*p* value	Preoperatively, *n* (%)	Postoperatively, *n* (%)	*p* value
Vaginal bulging						
Yes	1,017 (94)	223 (19)	<0.001	1,686 (96)	336 (19)	<0.001
No	62 (6)	972 (81)		73 (4)	1,613 (81)	
Satisfaction						
Satisfied	1,028 (89)				1,714 (89)	
Neither nor	90 (8)				134 (7)	
Dissatisfied	37 (3)				69 (4)	
Straining to void						
Never	545 (51)	999 (86)	<0.001	917 (53)	1,636 (85)	<0.001
Sometimes	171 (16)	89 (8)		307 (18)	179 (9)	
Daily	344 (33)	71 (6)		509 (29)	117 (6)	
Urinary incontinence						
Never	649 (60)	892 (76)	<0.001	1,069 (61)	1,443 (74)	<0.001
Sometimes	265 (25)	188 (16)		452 (26)	365 (19)	
Daily	165 (15)	97 (8)		241 (14)	143 (7)	
Urgency						
Never	331 (29)	764 (66)	<0.001	571 (33)	1,267 (67)	<0.001
Sometimes	302 (28)	249 (22)		534 (31)	432 (23)	
Daily	469 (43)	144 (12)		640 (38)	193 (10)	
Nocturia						
0–1 times per night	817 (74)	975 (82)	<0.001	1,337 (75)	1,607 (81)	<0.001
≥ 2 times per night	288 (26)	217 (18)		448 (25)	366 (19)	
Straining to defecate						
Never	744 (69)	927 (79)	<0.001	1,230 (71)	1,504 (78)	<0.001
Sometimes	246 (23)	203 (17)		408 (23)	345 (18)	
Daily	83 (8)	44 (4)		107 (6)	75 (4)	
Digitation						
Never	861 (82)	1,035 (90)	<0.001	1,436 (84)	1,728 (90)	<0.001
Sometimes	131 (12)	95 (8)		200 (12)	147 (8)	
Daily	63 (6)	28 (2)		72 (4)	34 (2)	
Sexually active						
No	632 (59)	577 (52)	<0.001	1,042 (59)	986 (53)	<0.001
Yes	448 (41)	534 (48)		715 (41)	879 (47)

**Table 3:** Comparison of patient-reported postoperative symptoms between groups

Symptoms	VH group^a^ (*n* = 1,195)	CA group (*n* = 1,979)	OR (95 % CI)^b, c^	*p* value
	Postoperatively, *n* (%)	Postoperatively, *n* (%)		
Vaginal bulging				
Yes	223 (19)	336 (19)	1.0 (0.7–1.3)	0.8
No	972 (81)	1,613 (81)		
Satisfaction				
Satisfied	1,028 (89)	1,714 (89)	1.1 (0.8–1.5)	0.6
Neither nor	90 (8)	134 (7)		
Dissatisfied	37 (3)	69 (4)		
Straining to void				
Never	999 (86)	1,636 (85)	0.8 (0.6–1.1)	0.2
Sometimes	89 (8)	179 (9)		
Daily	71 (6)	117 (6)		
Urinary incontinence				
Never	892 (76)	1,443 (74)	1.0 (0.8–1.3)	0.9
Sometimes	188 (16)	365 (19)		
Daily	97 (8)	143 (7)		
Urgency				
Never	764 (66)	1,267 (67)	0.9 (0.8–1.2)	0.6
Sometimes	249 (22)	432 (23)		
Daily	144 (12)	193 (10)		
Nocturia				
0–1 times per night	975 (82)	1,607 (81)	0.9 (0.6–1.2)	0.4
≥ 2 times per night	217 (18)	366 (19)		
Straining to defecate				
Never	927 (79)	1,504 (78)	1.1 (0.8–1.3)	1.0
Sometimes	203 (17)	345 (18)		
Daily	44 (4)	75 (4)		
Digitation				
Never	1,035 (90)	1,728 (90)	1.1 (0.9–1.5)	0.3
Sometimes	95 (8)	147 (8)		
Daily	28 (2)	34 (2)		
Sexually active				
No	577 (52)	986 (53)	1.0 (0.8–1.4)	0.8
Yes	534 (48)	879 (47)		

^a^Reference

^b^Modulated toward negative values

^c^Adjusted for age, parity, body mass index (BMI), degree of anterior and apical compartment prolapse, concomitant anterior colporrhaphy, symptoms at baseline, and menopausal status

The presence of a vaginal bulging sensation 1 year postoperatively was considered to be symptomatic recurrence and an analysis of the “cured prolapse” versus “recurrent prolapse” group was made to identify risk factors for recurrence. The two groups differed in that the patients with a symptomatic recurrence were somewhat younger (mean age ± SD; 61.5 ± 10.5 vs 63.4 ± 10.3, *p* = 0.001) and had a higher BMI (mean BMI ± SD; 26.2 ± 4.3 vs 25.7 ± 3.8, *p* = 0.04) compared with the patients with “cured prolapse.” There were no statistical differences when comparing the two groups considering the severity of prolapse in the apical or anterior compartment, functional status (ASA classification), parity, or whether or not concomitant anterior colporrhaphy was performed.

The primary outcome, the sensation of vaginal bulging 1 year postoperatively, was also evaluated in different subgroups (Table 4). Both techniques performed equally well in every subgroup analysis. The only exception was the subgroup without concomitant anterior colporrhaphy, in which cervical amputation was superior to vaginal hysterectomy.

**Table 4:** Primary outcome measure (vaginal bulging) 1 year after surgery

Sub groups	VH group^a^	CA group	OR (95%CI)^b^	*p* value
Uterine prolapse	Postoperatively, *n* (%)	Postoperatively, *n* (%)		
All patients stage I	*n *= 44	*n* = 134	0.7 (0.2–1.9)	0.5
No	35 (79)	100 (75)		
Yes	9 (21)	34 (25)		
All patients stage II	*n* = 381	*n* = 776	1.0 (0.7–1.5)	0.9
No	306 (80)	636 (82)		
Yes	75 (20)	140 (18)		
All patients stage III	*n* = 338	*n* = 459	1.1 (0.7–1.6)	0.8
No	281 (83)	380 (83)		
Yes	57 (17)	79 (17)		
All patients stage IV	*n* = 81	*n* = 49	0.9 (0.3–2.7)	0.8
No	66 (81)	38 (78)		
Yes	15 (19)	11 (22)		
All stages with concomitant AC	*n* = 1,013	*n* = 1,757	0.9 (0.6–1.1)	0.3
No	837 (83)	1,425 (81)		
Yes	176 (17)	332 (19)		
Stages II–III with concomitant AC	*n* = 595	*n* = 1,080	0.9 (0.7–1.2)	0.5
No	494 (83)	884 (82)		
Yes	101 (17)	196 (18)		
All stages with no concomitant AC	*n* = 181	*n* = 222	2.1 (1.1–4.3)	0.04
No	134 (74)	188 (85)		
Yes	47 (26)	34 (15)		
Stages II–III with no concomitant AC	*n* = 123	*n* = 155	2.5 (1.1–5.9)	0.04
No	92 (75)	132 (85)		
Yes	31 (25)	23 (15)		

*AC* anterior colporrhaphy

^a^Reference

^b^Adjusted for surgical method, age, parity, body mass index (BMI), degree of anterior and apical compartment prolapse (when appropriate), concomitant anterior colporrhaphy (when appropriate), symptoms at baseline, and menopausal status

### Adverse events

Adverse events are presented in Table 5. The rate of severe complications was significantly higher in the hysterectomy group than in the cervical amputation group, 23 out of 1,195 (1.9 %) vs 4 out of 1,979 (0.2 %), *p* < 0.001. The severe complications in the hysterectomy group were: intra-abdominal bleeding (*n* = 8), severe intra-abdominal infection or sepsis (*n* = 7), ureteric injuries (*n* = 4), bowel injuries (*n* = 2), myocardial infarction (*n* = 1), and severe complications related to the anesthesia (*n* = 1). The severe complications in the cervical amputation group consisted of: severe bleeding (*n* = 2) and severe infection (*n* = 2). There was no difference in the rate of mild to moderate complications between the groups, 146 out of 1,195 (12.2 %) in the vaginal hysterectomy group versus 246 out of 1,979 (12.4 %) in the cervical amputation group, *p* = 0.9.

**Table 5:** Perioperative data and complications

	VH group (*n* = 1,195)	CA group (*n* = 1,979)	*p* value
Complications, *n* (%)			
Mild and moderate	146 (12.2)	246 (12.4)	0.95
Severe	23 (1.9)	4 (0.2)	<0.001
Operation time (min) mean (±SD)	76.2 (±30.8)	50.0 (±21.2)	<0.001
Blood loss (ml) mean (±SD)	100.1 (±113.2)	44.6 (±64.5)	<0.001
Days at hospital mean (±SD)	1.7 (0.9)	0.8 (0.8)	<0.001
Days to normal ADL mean (±SD)	6.1 (±6.6)	4.8 (±6.3)	<0.001
Antibiotic prophylaxis, *n* (%)			
Yes	1,156 (97)	301 (15)	<0.001
No	39 (3)	1677 (85)	
Prophylaxis of venous thrombosis, *n* (%)			
Yes	1,040 (92)	866 (51)	<0.001
No	88 (8)	821 (49)	
Anesthesia, *n* (%)			
Intubation	597 (53)	620 (33)	<0.001
Other	538 (47)	1,268 (67)	

The hysterectomy group had a significantly longer mean duration of surgery (76.2 vs 50.0 min, *p* < 0.001) and greater mean amount of blood loss (100.1 vs 44.6 ml, *p* < 0.001) compared with the cervical amputation group. The hysterectomy group also received prophylactic antibiotics and prophylaxis against venous thrombosis to a greater extent, and they had a longer hospitalization (1.7 vs 0.8 days, *p* < 0.001) and a longer period until return to normal activities of daily living (6.1 vs 4.8 days, *p* < 0.001) than the cervical amputation group.

## Discussion

The results of this large, population-based register study indicate that cervical amputation performs equally as well as vaginal hysterectomy in the treatment of women with uterine prolapse. There was no difference in patient-reported symptom relief and satisfaction 1 year after surgery, but the rate of severe complications was significantly lower in the cervical amputation group. The findings corroborate those of previous studies comparing these two techniques [8, 13, 14]. In the subgroup of patients where no concomitant anterior colporrhaphy was performed, the cervical amputation group had a significantly lower symptomatic recurrence rate 1 year after surgery than the vaginal hysterectomy group, indicating that cervical amputation may be superior to vaginal hysterectomy in the treatment of apical descent.

In this study, cured prolapse was solely symptomatically defined, as we did not have information about postoperative POP-Q status. An absence of vaginal bulge symptoms strongly correlates with patient-reported improvement and treatment success [17]. Using the definition described above, treatment success was similar (81 %) in each group. In a study by Thys et al. comparing the Manchester Fothergill (MF) procedure with vaginal hysterectomy (VH), the objective recurrence rates were 18 % in the MF group and 19 % in the VH group with a median follow-up time of 75 months and there were no differences in POP-related symptoms postoperatively [13]. De Boer et al. compared the modified Manchester procedure (cervical amputation with uterosacral ligament plication) with vaginal hysterectomy. Both procedures performed excellently in the middle compartment [14]. In a review article from 2009 by Dietz et al., the anatomical cure rate in apical support ranged between 93 and 100 % in the Manchester procedure group.

In the present study, the preoperative degree of uterine prolapse was more pronounced in the vaginal hysterectomy group compared with the cervical amputation group (Table 1). This may reflect a general conception that suspension of the vaginal cuff is needed for proper repair of advanced apical prolapse. However, similar treatment results persisted after a multivariate regression analysis adjusting for preoperative POP-Q stage, among other variables. When comparing the results of vaginal hysterectomy with cervical amputation in each subgroup of preoperative POP-Q stage, there were no significant differences. Hence, cervical amputation with ligament attachment can also be considered in advanced uterine prolapse.

Uterine prolapse is often associated with co-existing prolapse in the anterior vaginal wall [18], which is reflected in this study, where the vast majority of patients had concomitant anterior vaginal wall repair. This study cannot answer the question: does the presence of prolapse symptoms postoperatively represent a failure in the apical or the anterior compartment? In both groups, significant improvement was seen in all self-reported bladder dysfunction symptoms, as expected after an anterior colporrhaphy [19, 20]. It was notable, however, that self-reported symptoms of obstructive defecation also became significantly less frequent, indicating that symptoms of uterovaginal prolapse do not necessarily correlate with compartment-specific defects [21].

Increasing age and excess weight are established risk factors for pelvic organ prolapse [1]. In the present study, women with a lower BMI had less symptomatic recurrence than the group with higher BMI. However, symptomatic recurrence correlated inversely with age and the preoperative grade of prolapse did not affect the risk of recurrence. One could speculate that younger women might be more physically active and thus performing “heavy activities” postoperatively to a greater extent, which increases the risk of recurrence.

Various surgical methods have been developed to improve the outcome after surgery for apical prolapse. Sacrocolpopexy and sacrospinous fixation can be performed not only in the treatment of vaginal vault prolapse, but also as uterus-sparing techniques. Open abdominal sacrocolpopexy is the most successful method in the surgical treatment of apical prolapse regarding recurrence rates, but the procedure is associated with an increased length of hospital stay, analgesic requirements, and costs compared with transvaginal procedures [2, 22]. Laparoscopic and robot-assisted sacrocolpopexies also provide excellent short- to medium-term reconstructive outcomes for patients with POP, but involve a shorter recovery time than with open procedures [23]. These techniques, though, can only partly replace the traditional ones, as they require both high-technology operating facilities and experienced surgeons. Their cost-effectiveness is currently unclear [23]. The use of mesh in prolapse surgery has reduced recurrence rates and has therefore been used more frequently over the last decade. However, the benefits must be weighed against the disadvantages, such as mesh erosion (5–10 %) and dyspareunia [24–26]. Sacrospinous hysteropexy is another uterus-preserving technique that is aimed at fixing the uterus to the sacrospinous ligament—most commonly to the right side to prevent lesions of the rectum. A newly published randomized trial by Detollenaere et al. showed equal outcomes of sacrospinous hysteropexy compared with vaginal hysterectomy [11].

Strengths of this study include prospective data collection and a large study population treated in a routine medical care setting. The operating clinics vary from large-scale teaching hospitals to smaller private practitioners and the geographical distribution of the patients is wide, which also increases the external validity. The overall response rate of 78 % must be considered an acceptable figure in a questionnaire-based study and the possibility of response bias is relatively low [27].

The lack of objective measures postoperatively is a limitation of our study, as this could have provided relevant information about prolapse symptoms in relation to anatomical outcome after uterine prolapse surgery. However, the aim of POP surgery is symptom relief regardless of postoperative anatomy. The register data provide no information about the position of the isthmus and cervical length. Therefore, it is not possible to evaluate the influence of possible cervix elongation on the choice of surgical method. The literature contains no clear definition of cervical elongation. Some authors suggest a definition including the corpus uteri/cervix ratio (CCR) of < 1.5 [28, 29]. Using that definition, Mothes et al. found that cervical elongation was present in 97.6 % of patients undergoing hysterectomy due to objective and symptomatic uterine POP stage II–IV, which would suggest that a considerable amount of the patients in the present study might have had some degree of cervical elongation [28]. Suspension of the vaginal apex or cervical end is a standard procedure in vaginal hysterectomy and cervical amputation, but the methods vary. The database does not contain information regarding whether a suspension of the cuff/stump was performed and details of the suspension techniques are not registered. It is, in our opinion, highly important to perform some kind of cuff suspension to restore apical support, as it is not the hysterectomy alone that corrects the prolapse.

The follow-up time of 1 year allows us to evaluate only short-term outcomes. One long-term disadvantage of cervical amputation is the possibility of cervical stenosis and thus complications such as hematometra and difficulties in diagnosing endometrial pathological conditions using histological samples. These complications are not possible to evaluate in the present study.

Another limitation of this study is that the patient questionnaires used in the register database are not validated POP questionnaires. However, the primary outcome question regarding vaginal bulging sensation has been validated.

In conclusion, this study shows no difference between cervical amputation and vaginal hysterectomy in symptomatic outcomes or patient satisfaction in the treatment of uterine prolapse 1 year after surgery. Cervical amputation is a less invasive procedure with a short operation time, reduced blood loss, low complication rates, and fast postoperative recovery time compared with vaginal hysterectomy. The use of prophylactic antibiotics and low molecular weight heparin can also be reduced when performing a cervical amputation rather than a vaginal hysterectomy. Based on these outcomes, we suggest that cervical amputation is a reliable option with few complications in the treatment of women with uterine prolapse, when no other indication for removal of the uterus exists. Randomized controlled trials with long-term follow-up are still needed to evaluate a comparison of cervical amputation not only with vaginal hysterectomy, but also with other uterine-preserving procedures.
